# The complete mitochondrial genome of bobo croaker *Pseudotolithus elongatus* (Perciformes: Sciaenidae)

**DOI:** 10.1080/23802359.2019.1667920

**Published:** 2019-09-24

**Authors:** Jong-Oh Kim, Yong Bae Seo, Jiyoung Shin, Ji-Young Yang, Gun-Do Kim

**Affiliations:** a Institute of Marine Biotechnology, Pukyong National University, Busan, The Republic of Korea;; b Department of Food Science & Technology, Pukyong National University, Busan, The Republic of Korea;; c Department of Microbiology, Pukyong National University, Busan, The Republic of Korea

**Keywords:** *Pseudotolithus elongates*, Sciaenidae, mitochondrion genome, phylogenetic analysis

## Abstract

The complete mitochondrial genome of *Pseudotolithus elongatus* (Perciformes: Sciaenidae) is determined based on NGS technology. The assembled mitogenome is a 16,497 bp in length containing a typical set of the 13 protein-coding genes, 22 tRNAs, 2 rRNA genes, and the 1 putative control region. The overall base composition is A (27.8%), T (25.3%), G (16.1%), and C (30.8%) with an A-T content of 53.1%. The phylogenetic analysis of 36 mitogenomes from the GenBank indicated that *P. elongatus* is closely related to the *Aplodinotus grunnien*s. This mitogenome information of the *P. elongatus* would be useful to understand evolutionary and phylogenetic analysis of the family Sciaenidae fishes.

Bobo croaker *Pseudotolithus elongatus*, belongs to the family Sciaenidae, is widely distributed in the west coast of Africa (Vreven and Snoeks 2008). It is one of the significant imported fish in the Korean fisheries market and gradually increasing its imports to Korea. Recently, economically motivated adulteration (EMA) cases – cheating by other species – are occurring frequently because these imported fish are difficult to distinguish from other similar fish by their morphological features. Although there are some publications on biological, physiological characteristics (Nawa 1985; Chao and Trewavas 1990; Abowei et al. 2008), the genetic information is not sufficient till date. And so, this study aims to provide the genetic information for *P. elongatus* by analyzing the mitochondrion genome sequence.

The specimen in the frozen state caught off the Guinea coast (9°34'04.6''N 13°35'34.3''W) was purchased from an international fish importer in Korea. Before starting the mitogenome sequencing, its morphology and *COI* gene sequence analysis were accompanied for a precision identification. The specimen was stored at Department of Food Engineering, Pukyong National University (Sample no. MO00171429) under the research grant from Ministry of Food and Drug Safety. The total genomic DNA was extracted from the muscle tissue and DNA library was constructed with the TruSeq Nano DNA Sample Preparation kit (Illumina, San Diego, CA). The library was sequenced by Illumina NextSeq 500 sequencing platform (150 paired-ends). The obtained raw reads were trimmed and the low-quality reads (Q < 20) were removed. The cleaned sequences were mapped to previously reported mitogenome sequences of Sciaenidae (Zhao et al. 2012) using Geneious 11.1.3 (Kearse et al. 2012) and annotation was performed by MitoFish pipeline (Sato et al. 2018). In addition, phylogenetic analysis was performed using the maximum-likelihood method with 1000 bootstraps in MEGA X (Kumar et al. 2018).

The assembled mitogenome is a 16,497 bp in length (GenBank accession no. MN251863) containing a typical set of the 13 protein-coding genes (PCGs), 22 tRNAs, 2 rRNA genes, and the 1 putative control region. The overall base composition of *P. elongatus* is A (27.8%), T (25.3%), G (16.1%), and C (30.8%), with A-T content of 53.1%. Most encoding genes are located on the H-strand except *ND6* and 8 tRNAs (*Gln*, *Ala*, *Asn*, *Cys*, *Tyr*, *Ser*, *Glu*, *Pro*) genes. All PCGs use typical ATG start codon while incomplete stop codons were identified in seven genes including *ND2*, *COX2*, *ATP6*, *COX3*, *ND3*, *ND4*, *CYTB*.

The phylogenetic analysis of 36 mitogenomes from GenBank indicated that *P. elongatus* is closely related to the *Aplodinotus grunniens*. The complete mitogenome of *P. elongatus* is the second announced mitogenome within the genus *Pseudotolithus* after the mitogenome of *Pseudotolithu senegallus* (Accession No. MH995529). However, these two species show a genetic distance greater than other species in Sciaenidae (Figure 1). Thus, for a more detailed understanding of relationship for the genus *Pseudotolithus*, further sampling and sequencing researches are required. This mitogenome information of *P. elongatus* will be useful to elucidate the phylogenetic relationships of the genus *Pseudotolithus* and related genera.

**Figure 1.**
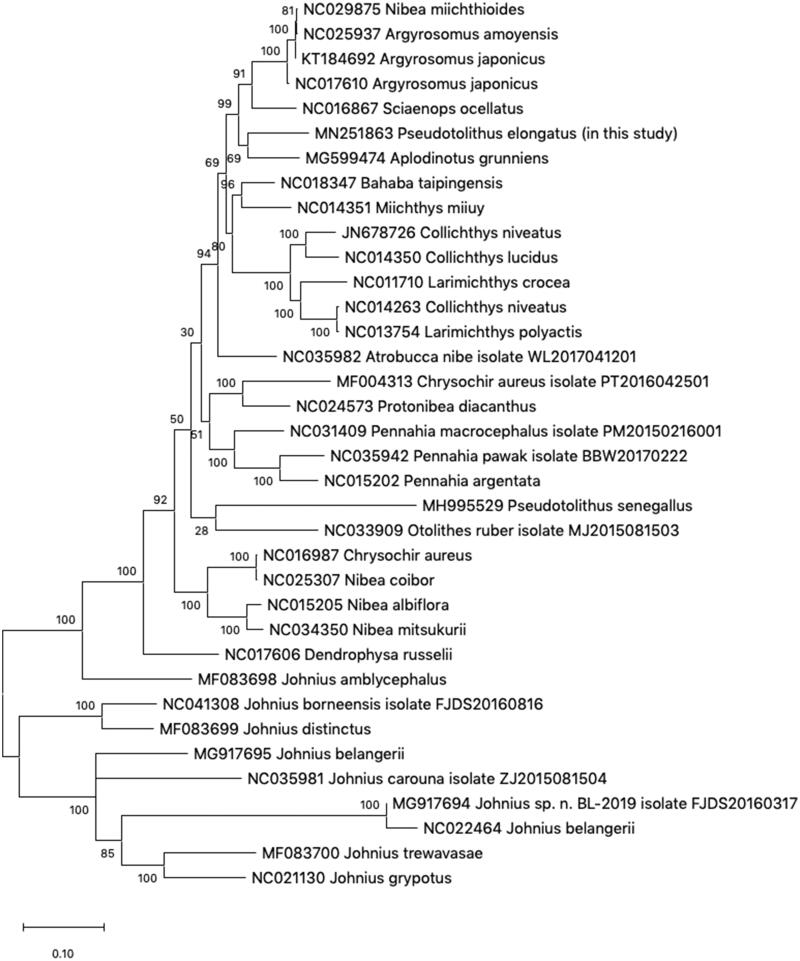
Phylogenetic tree of 36 species in family Sciaenidae. The complete mitogenomes were obtained from GenBank and phylogenetic analysis was conducted by maximum-likelihood method with 1000 bootstraps. The percentage at each node is the bootstrap probability.
